# Chloroplast Distribution in the Stems of 23 Eucalypt Species

**DOI:** 10.3390/plants9121814

**Published:** 2020-12-21

**Authors:** Geoffrey E. Burrows, Celia Connor

**Affiliations:** School of Agricultural and Wine Sciences, Charles Sturt University, Locked Bag 588, Wagga Wagga, NSW 2678, Australia; cconnor@csu.edu.au

**Keywords:** *Angophora*, bark, corticular photosynthesis, *Corymbia*, *Eucalyptus*, fluorescence, phellem, stem photosynthesis, wood photosynthesis

## Abstract

Small diameter branchlets and smooth barked stems and branches of most woody plants have chloroplasts. While the stems of several eucalypt species have been shown to photosynthesise, the distribution of chloroplasts has not been investigated in detail. The distribution of chloroplasts in branchlets (23 species) and larger diameter stems and branches with smooth bark (14 species) was investigated in a wide range of eucalypts (species of *Angophora*, *Corymbia* and *Eucalyptus*) using fresh hand sections and a combination of bright field and fluorescence microscopy. All species had abundant stem chloroplasts. In both small and large diameter stems, the greatest concentration of chloroplasts was in a narrow band (usually 100–300 μm thick) immediately beneath the epidermis or phellem. Deeper chloroplasts were present but at a lower density due to abundant fibres and sclereids. In general, chloroplasts were found at greater depths in small diameter stems, often being present in the secondary xylem rays and the pith. The cells of the chlorenchyma band were small, rounded and densely packed, and unlike leaf mesophyll. A high density of chloroplasts was found just beneath the phellem of large diameter stems. These trees gave no external indication that green tissues were present just below the phellem. In these species, a thick phellem was not present to protect the inner living bark. Along with the chlorenchyma, the outer bark also had a high density of fibres and sclereids. These sclerenchyma cells probably disrupted a greater abundance and a more organised arrangement of the cells containing chloroplasts. This shows a possible trade-off between photosynthesis and the typical bark functions of protection and mechanical strength.

## 1. Introduction

Leaves, specifically the lamina or blade, are the primary site of photosynthesis for most species. For some plants, modified petioles (phyllodes) or modified stems (cladodes) are the primary photosynthetic organs. Nonetheless, a wide range of parts of seedlings and mature plants are green and photosynthetic, e.g., hypocotyl, cotyledons, flowers, fruits, and roots [1]. In addition, small and large diameter stems, although not obviously green, can have numerous chloroplasts in the living bark cells as long as the phellem or rhytidome are relatively thin. Nearly all woody plants carry out photosynthesis in their twigs, but far fewer do so in larger diameters [2,3].

Several ways of classifying photosynthesis in stems have been proposed. There are structural or anatomical classifications (e.g., [4,5,6,7]) (see also tables in [2,8]), such as:

1. CAM plants (stem succulents, often with cladodes).
2. Stem photosynthesis—green stems with epidermis (no or delayed periderm development) and a high stomatal density [9], with some species having a well-developed palisade layer (e.g., [10]). They are mainly herbaceous plants but not exclusively and include desert shrubs [10] and early successional legumes (references in [11]).
3. Corticular or bark photosynthesis in woody plants after periderm development. Ávila et al. [9] indicate that this type of structure should be termed ‘cortical photosynthesis’. In stems of trees and shrubs, the zone between the periderm and the secondary phloem is often referred to as ‘cortex’. However, the cortex can be defined as a primary ground tissue between the epidermis and vascular bundles in a stem [12]. Thus, ‘secondary cortex’ [13] and ‘pseudo-cortex’ [14] have also been used for this zone. Rosell [15,16] considers that these cells are initially derived from the shoot apical meristem but then divide radially, thus are primary in origin (not derived from the vascular or cork cambia). Anatomical aspects of corticular photosynthesis have been more commonly investigated in small diameter/young stems (Table 1). Chloroplasts have been recorded in current year shoots to trunks 60 cm in diameter. In the few studies where both small and large diameters were examined, chloroplasts were found at greater depths in the smaller diameters (e.g., [17]). This may be related to light being conducted in the axial system of vessels, tracheids, and fibres (e.g., [18]). As could be expected, the density of chloroplasts decreases as phellem thickness increases (e.g., [3]).
4. Wood or woody tissue photosynthesis usually occurs in the xylem ray parenchyma cells and can also extend into the pith in small diameter stems.

Ávila et al. [9] proposed a physiological/anatomical classification: stem net photosynthesis (SNP) where CO_2_ from the atmosphere enters the stem via stomata (equivalent to stem photosynthesis) and stem recycling photosynthesis (SRP) where the chloroplasts fix CO_2_ released from respiration of living stem cells (equivalent to corticular and woody tissue photosynthesis). In the latter, a periderm is present and this has high resistance to gas diffusion and is a barrier to light [9]. Internal transport of CO_2_ from roots to stems in the xylem transpiration stream can also occur [19].

In species with SNP, cells can be maximised for photosynthesis (i.e., presence of palisade, [10]). Bark has multiple functions including protection, storage, mechanical support, translocation of photosynthates and photosynthesis [3,16]. It is probable, especially in plants with well-developed leaves, that cell types and arrangement in the bark would be maximised for protection and mechanical strength rather than photosynthesis. As noted, in species with SRP, the periderm blocks CO_2_ from entering and leaving the stem and thus the chloroplasts refix the CO_2_ released by living cells in the bark. SRP can make a significant contribution to the overall carbon balance, water use efficiency [20], and drought tolerance [21]. Cernusak and Cheesman [11] note that with thin phellem, there is a possible trade-off between protection of stems from fire and high rates of SRP.

There are over 800 species of eucalypts (species of *Angophora*, *Corymbia* and *Eucalyptus*) [22]. They have what is probably the greatest diversity of bark types in a single group of trees. Bark types vary from entirely smooth (gum), smooth with scribbles, ironbark, stringybark, bloodwood, box, minniritchi, tessellated, as well as half-barks (half-butts) that have a rough bark on the lower half of the tree and smooth bark on the upper half. All eucalypt species have green or reddish branchlets covered by an epidermis and cuticle. They then develop relatively smooth bark on their small diameter (e.g., 0.5–2 cm) stems, before developing the more distinctive bark types as stem diameter increases. Many eucalypt species are smooth barked throughout their ontogeny. The bark of about 50% of eucalypt species is wholly smooth over the main stem and major branches and in 75% of species it is smooth over canopy branches [20]. Gillison ([23] p. 41) notes that species with green stem or cortical photosynthesis are evident in holarctic and boreal regions but most species occur in the tropics “in particular among the gum-barked eucalypts of Australia”. Along with a large percentage of species with smooth bark, most eucalypt species have relatively narrow (lanceolate) leaves that hang vertically, thus relatively high light intensities are intercepted by the trunk and branches. In addition, there is a high proportion of half-butt or half-bark (where the basal thicker dead bark can provide fire protection while the upper thin phellem might allow sufficient light to enter a branch for photosynthesis) eucalypt species, especially in the more arid areas of Australia. SRP is associated with increased whole plant water-use efficiency and reduced drought effects as CO_2_ is refixed with minimal water loss [11]. This is important for this widely distributed group of three genera in an arid continent being affected by climate change and longer fire periods. As noted, plants with SNP (long lived green stems) can have stem stomatal densities similar to those found in leaves (e.g., [10]). Young stems of tree species usually have very low stomatal densities (e.g., [6]). As recently formed eucalypt shoots are greenish (or reddish), it was of interest to quantify stomatal density as this would give some indication of whether SNP or SRP (or a combination of both) occurred.

Physiological studies of SRP have been made in eucalypt species such as *C. citriodora* [24], probably *E. dunnii* (although the species name is given as *EuCahetus dunnii*) [25], *E. globulus* [26,27], *E. grandis* × *E. urophylla* [24,28], *E. miniata* [20], *E. nitens* [29], and *E. saligna* [30,31]. All these species were smooth barked (gums), except for *E. miniata* (half-butt), where photosynthesis research centered on the smooth upper half of the trees.

Few studies of chloroplast distribution in eucalypt stems have been made (e.g., [3,20,24,28,32,33,34,35,36,37]). Mostly these studies only recorded that greenish stem tissues were observed. Chen et al. [24] studied four tree species with SRP, two eucalypt species and two other species not in the Myrtaceae. They noted the eucalypts’ cortical green tissue was about 2 mm thick, while in the other species, this tissue was only about 0.5 mm thick. The only anatomical study was that of Mishra et al. [37] where the autofluorescence of chloroplasts in secondary xylem ray parenchyma of two-year-old plants of *E. bosistoana* was recorded.

Given that there have been several physiological studies of SRP in eucalypts, but only one detailed study of chloroplast distribution in the stems of a single eucalypt species, this study concentrates on the anatomical aspects of SRP in this important group of trees. When green tissues are found just beneath the phellem of stems, it is usually assumed that this colouration is from chloroplasts (e.g., Rosell [3]), that these chloroplasts will be able to photosynthesise and this photosynthesis would be SNP. We also make this assumption.

It is clear that SRP is a key aspect of eucalypt carbon balance (e.g., [20]) and this can impact drought tolerance. To better understand these features, it is important to survey the presence of chloroplasts in the stems of a wide taxonomic diversity of eucalypt species, in a wide range of stem diameters, and to quantify the location of the chloroplasts in those stems. In addition, it has been suggested that there may be a trade-off between fire protection and photosynthesis in larger diameter trunks and branches. An anatomical study of chloroplast distribution is essential to evaluate any trade-off. We studied chloroplast distribution in stems from a wide range of eucalypt species and hypothesised that:

i) given the findings of the studies in Table 1, chloroplasts would be found in all the small diameter branches as well as large diameter branches and trunks of smooth barked stems,
ii) given the wide taxonomic diversity of species examined, different chloroplast arrangements would be recorded,
iii) given that light appears to be conducted more effectively in small diameter stems, chloroplasts would be found at greater depth in smaller diameter stems than larger diameter ones,
iv) the youngest stems (no periderm) would have stomata but they would be at a low density compared to adjacent leaves, and
v) given that the species examined had thin phellem (provides little protection), the bark inside this layer would be optimised for defence and mechanical support rather than photosynthesis.

## 2. Results

### 2.1. External Morphology

**Figure 1:**
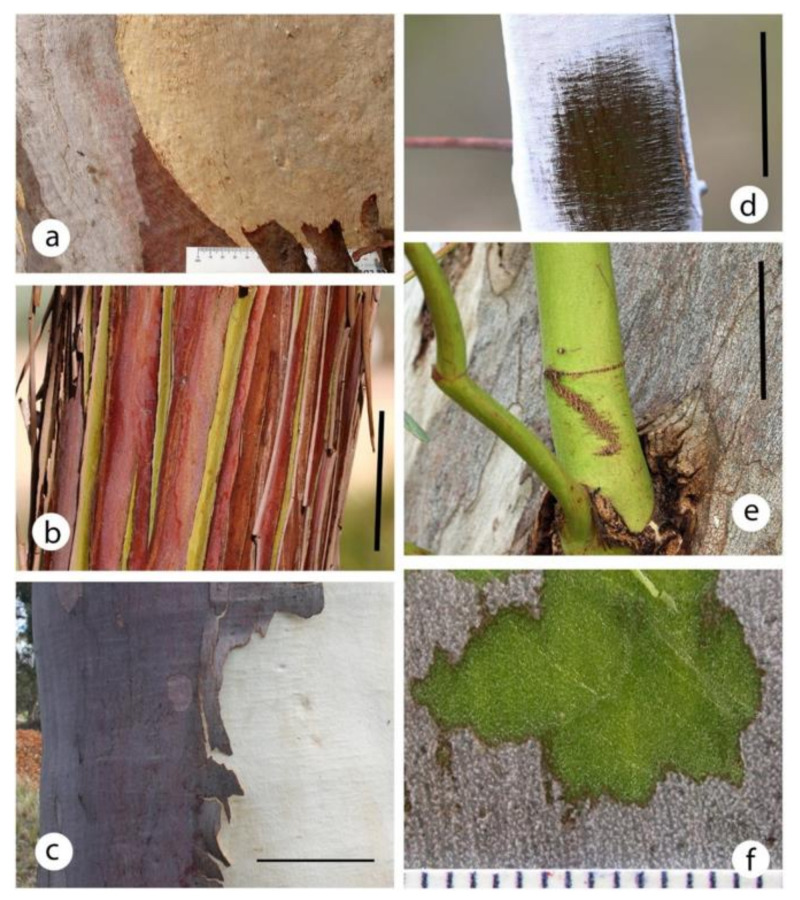
Bark surfaces of various eucalypt species showing the wide diversity of bark morphologies and the delayed bark formation that occurs in some species. (**a**) Surface of the main trunk of a *Eucalyptus blakelyi* tree showing that the phellem gets darker and slightly thicker as it ages and is shed in irregularly shaped patches. (**b**) Minniritchi bark on a main stem of *Eucalyptus caesia*. Note the alternating strips of green and brownish red bark. Scale 5 cm. (**c**) Trunk (40 cm diameter) of *Corymbia citriodora* in early summer during the bark shedding phase. Note that when the bark was initially formed (right hand side), it was almost white, but when shed a year later, it was much darker. Scale 10 cm. (**d**) Young branch of *Eucalyptus caesia* showing the highly reflective glaucous layer that has been partially rubbed away to reveal the epidermis and greenish stem. Scale 2 cm. (**e**) Part of the trunk of a large *Eucalyptus cladocalyx* tree that had formed rapidly growing epicormic shoots after the tree had been extensively pruned. Note the smooth gum bark on the large diameter bole and the bright green epicormic shoots. The largest shoot still had an epidermis and stomata (the minute white spots) although it was over 2 cm diameter. Scale 5 cm. (**f**) Bark of *Corymbia citriodora* with some of the thin (<100 μm thick) phellem removed with a scalpel, revealing the dark green tissues directly below the phellem. Scale in mm.

As the phellem of rough barks such as iron and stringy barks can be centimetres thick, thus totally excluding light from the living bark tissues, this study was of smooth barked species (Figure 1). Smooth bark was maintained by shedding of the periderm, either on an annual basis (Figure 1c) or in sections over time (Figure 1a,c). Although the phellem was generally very thin in almost all species with periderm formation, the surface of the bark was not green or greenish. The only exception was the sections of the minniritchi bark of *Eucalyptus caesia* that had recently peeled (Figure 1b). In the studied species, a gentle scape of the phellem cells revealed the bright green tissues of the outer bark (Figure 1f).

### 2.2. Small Diameter Stems Without Periderm

**Figure 2:**
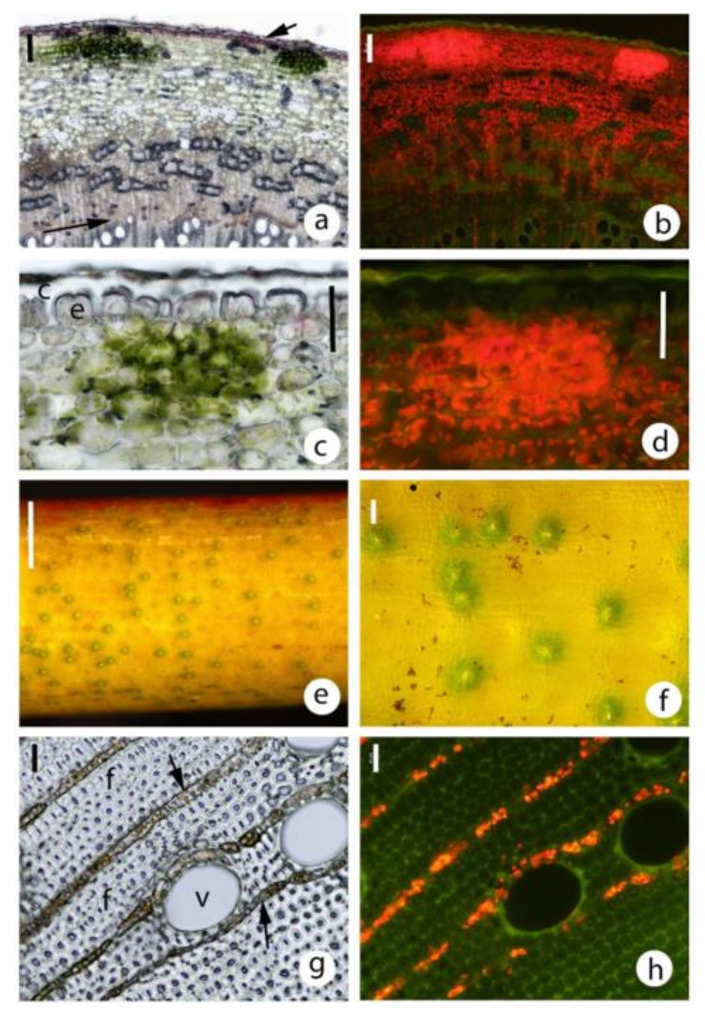
Chloroplast distribution in small diameter eucalypt stems. Note in (**a**–**f**), the much greater chloroplast density in the vicinity of the low density stomata. All sections (**a**–**d**,**g**,**h**) are transverse. (**a**) *Corymbia ficifolia* stem 3.5 mm diameter, bright field illumination, showing a green band between the epidermis (short arrow) and the secondary phloem. Note the two darker green regions just beneath the epidermis that were probably associated with stomata. vascular cambium, long arrow. Scale 100 μm. (**b**) as per (**a**) but showing chloroplast auto-fluorescence. Scale 100 μm. (**c**) *Eucalyptus sideroxylon*, 3 mm diameter shoot, showing detail of clustered chloroplasts associated with a stoma (bright field illumination). c, cuticle; e, epidermis. Scale 50 μm. (**d**) as per (**c**) but showing chloroplast auto-fluorescence. Scale 50 μm. (**e**) Segment of small diameter *Eucalyptus sideroxylon* branch showing the distribution of stomata and associated areas of higher chloroplast density. Note the wide variation in stomatal density. This stem had, on average, about 5 stomata/mm^2^. Scale 1 mm. (**f**) detail of a stem like that in (**e**). Note the outline of the typical epidermal cells and the guard cells. Scale 100 μm. (**g**) Transverse section of the secondary xylem in a *Eucalyptus cladocalyx* stem 13 mm diameter. Note the presence of starch grains and chloroplasts that were almost entirely restricted to the ray parenchyma cells. f, fibres; v, vessels; arrow, xylem ray. Scale 20 μm. (**h**) as per (**g**) but showing chloroplast auto-fluorescence. Scale 20 μm.

**Figure 3:**
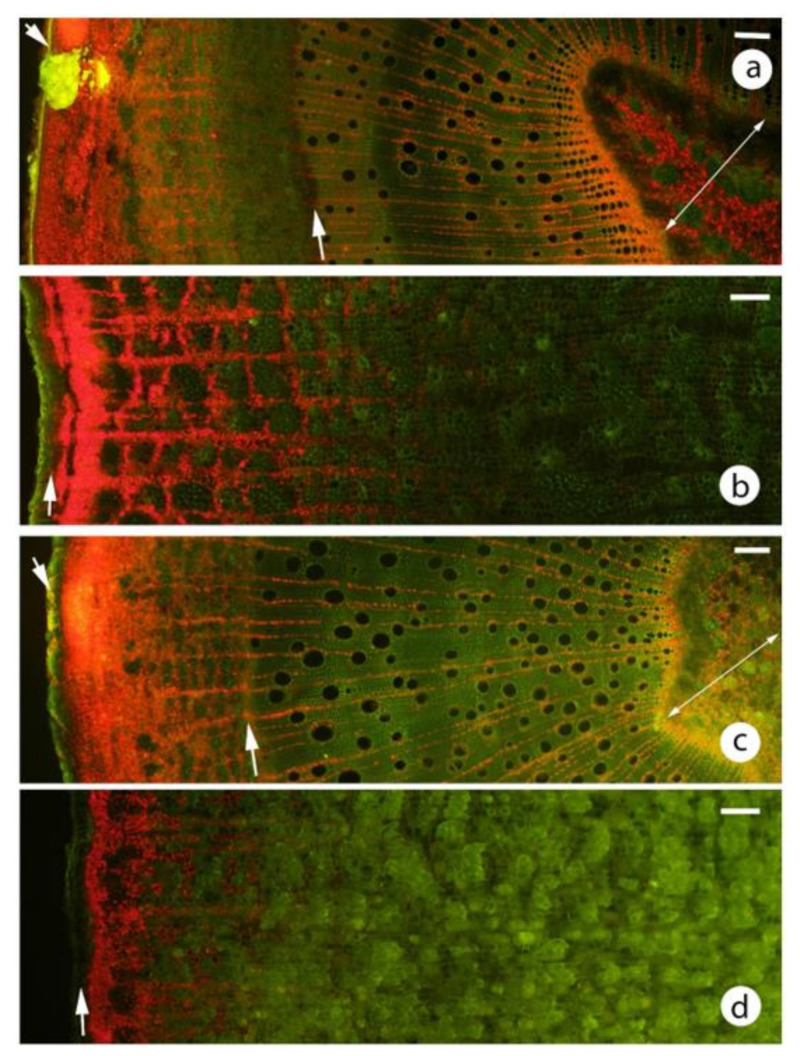
Differences in depth of chloroplasts in small and large diameter eucalypt stems. Red and green indicate the location of the chloroplasts and sclerenchyma, respectively. Note that in the small diameter stems chloroplasts were present from immediately beneath the epidermis to the pith, while in the large diameter stems they were only present in the outer bark. (**a**,**b**) *Eucalyptus globulus*, (**c**) and (**d**) *Eucalyptus cladocalyx*. All scale bars 100 μm. (**a**) 6 mm diameter. The bright yellow area near the epidermis was possibly the contents of a resin duct. short arrow, epidermis; long arrow, vascular cambium; line, pith. (**b**) 50 cm diameter. arrow, phellogen. (**c**) 4 mm diameter. Labelling as per (**a**). (**d**) 30 cm diameter. arrow, phellogen.

Transverse sections showed that almost all regions of the stems, except the epidermis (i.e., cortex, secondary phloem, secondary xylem, pith) have chloroplasts (Figure 2 and Figure 3a,c). In the secondary xylem, chloroplasts were restricted to the ray parenchyma cells (along with starch grains) (Figure 2a,b,g,h and Figure 3a,c), thus with fluorescence microscopy, radiating lines of red chloroplasts were visible. The highest density of chloroplasts was in a 100–400 μm wide cortical band just under the epidermis (Figure 2a,b and Figure 3a,c). The chloroplasts were in compact, rounded cortical cells with little intercellular airspace (Figure 2a–d), not in elongated palisade-type cells. Chloroplasts in the inner secondary xylem and pith could be more than 1500 μm in from the epidermis (Figure 3a,c).

In most species, small circular to oval areas were observed in the outer cortex that were a brighter green in bright field microscopy (Figure 2a,c) and much brighter red in fluorescence microscopy (Figure 2b,d) than the general distribution of chloroplasts. Examination of the stem surface with a stereomicroscope revealed the presence of scattered small darker green areas (Figure 2e), but their conspicuousness depended on the species and the area of the stem examined. High power stereomicroscopy and epidermal replicas indicated that the green areas were below stomata (Figure 2f). Stomatal density was much lower on stems than leaves (Table 2) and much more variable in density (Figure 2e) (relative standard deviation 17% leaves, 59% stems). These darker green areas were a small volume of the cortex (Figure 2e,f).

The small diameter stems of some species (*E. caesia*, *E. kruseana*, *E. macrocarpa*) were covered by a dense glaucous wax layer. These stems were almost pure white (Figure 1d) but still had substantial chloroplast development.

### 2.3. Large Diameter Stems

**Figure 4:**
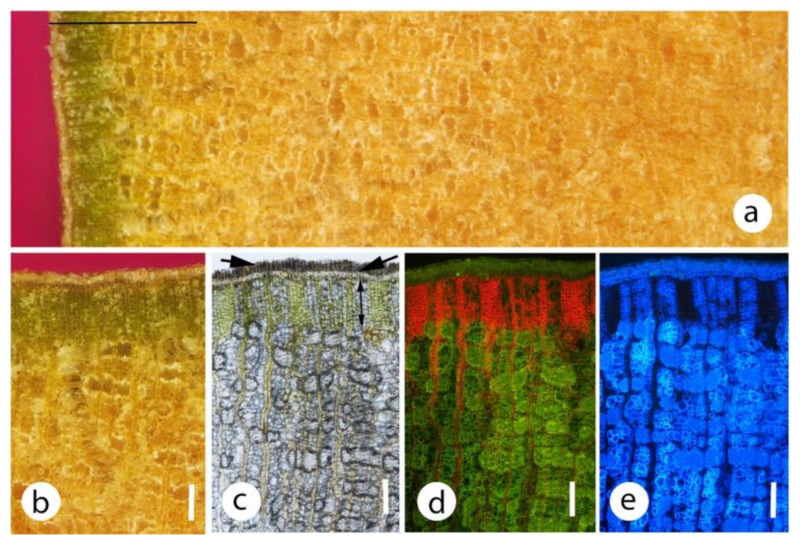
Various images, either transverse section or transverse plane, of the bark from a 40 cm diameter trunk (bark thickness 9–10 mm) of *Corymbia citriodora*. Note the greater density of chloroplasts in the phelloderm. Chloroplasts occurred deeper (mainly in the ray-like parenchyma cells) but overall density was much lower. All images were of the same small block of bark. (**a**) Outer 5 mm bark thickness observed with a stereo microscope. Note the thin phellem and green layer directly beneath the phellem. Scale 1000 μm. (**b**) Area of (**a**) at higher magnification. Note that the cells in the green layer were in files of cells perpendicular to the surface. Scale 200 μm. (**c**) Hand cut section of outer bark observed with a compound microscope and bright field illumination. short arrow, phellem; long arrow, phellogen; line, phelloderm. Scale 200 μm. (**d**) as per (**c**) but fluorescence microscopy. Note that from the red auto-fluorescence, most of the chloroplasts were in a 300 μm deep band immediately beneath the phellem, although deeper chloroplasts were found in ray-like structures. Scale 200 μm. (**e**) As per (**d**), except UV light shows the high density of fibres and sclereids (blue fluorescence) especially below the chlorenchyma band. Scale 200 μm.

In the 13 species studied, the phellem was thin (≤100 μm) (Table 3) and in *C. citriodora* was very thin (<50 μm) in all stem diameters studied (2–45 cm) (Figure 1f). The greatest density of chloroplasts was usually in a narrow band (80–400 μm) (Table 3) immediately beneath the phellem (Figure 3b,d). Less abundant chloroplasts were found 600–1800 μm further into the bark, usually in parenchyma cells of the secondary phloem or secondary cortex (Figure 3b,d or Figure 4b,c). The outer region of the living part of the bark (the phellogen and outer secondary phloem or secondary cortex) had a large proportion of thick walled, lignified cells (fibres and sclereids) (Figure 3b,d or Figure 4e). The sclereids usually had relatively thin walls. Both cell types were usually devoid of chloroplasts in their lumens. Some sclerenchyma cells were present in the chlorenchyma band but were far more abundant in the remainder of the bark Figure 3b,d or Figure 4e).

**Figure 5:**
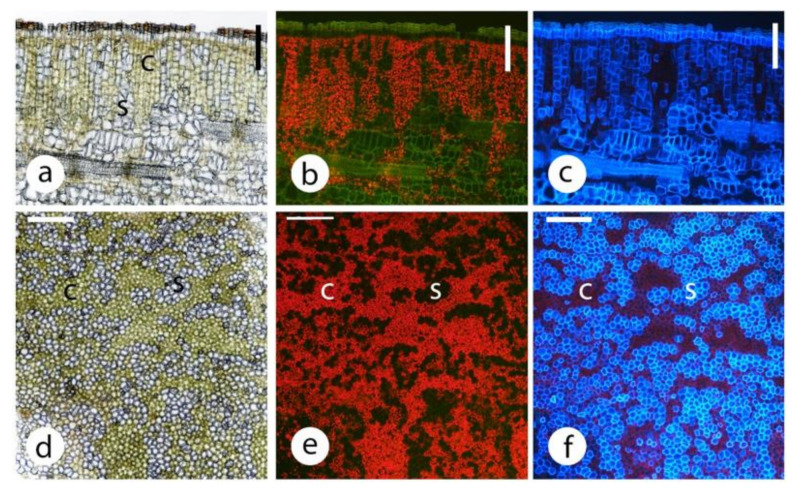
Bark from the main stem of *Corymbia citriodora*, transverse (**a**–**c**) and tangential longitudinally sectioned (**d**–**f**). c, chlorenchyma; s, sclereids. Sections were imaged with white (**a**,**d**), blue (**b**,**e**) and UV (**c**,**f**) light. Note that the phellem was very thin and the greatest concentration of chloroplasts was in the phelloderm cells. Note also that while the phelloderm cells are exposed to relatively high light intensities, many of these cells have differentiated into sclereids. The tangential longitudinal sections show the chlorenchyma and sclerenchyma were apparently arranged in random, but relatively discrete, tissue groupings. Scales 200 μm.

In *C. citriodora* and the closely related *C. maculata,* a well defined dark green band was present directly beneath the phellem (Figure 4a,b) and under a stereo microscope this band had a palisade-like appearance. Sectioning showed these cells were probably phelloderm, about 10–15 cells deep (Figure 4c–e). Most cells of this band contained chloroplasts, but relatively small and relatively thin-walled sclereids were also present (Figure 4e and Figure 5). Tangential longitudinal sections of the band (paradermal-type sections) showed that the chlorenchyma and sclereids were usually segregated into different blocks of tissues rather than being a homogeneous mix (Figure 5). The chlorenchyma cells were thin walled, with little intercellular air space development. Generally, a distinct differentiation was present between the two cell types but some sclereids with chloroplasts were observed.

**Figure 6:**
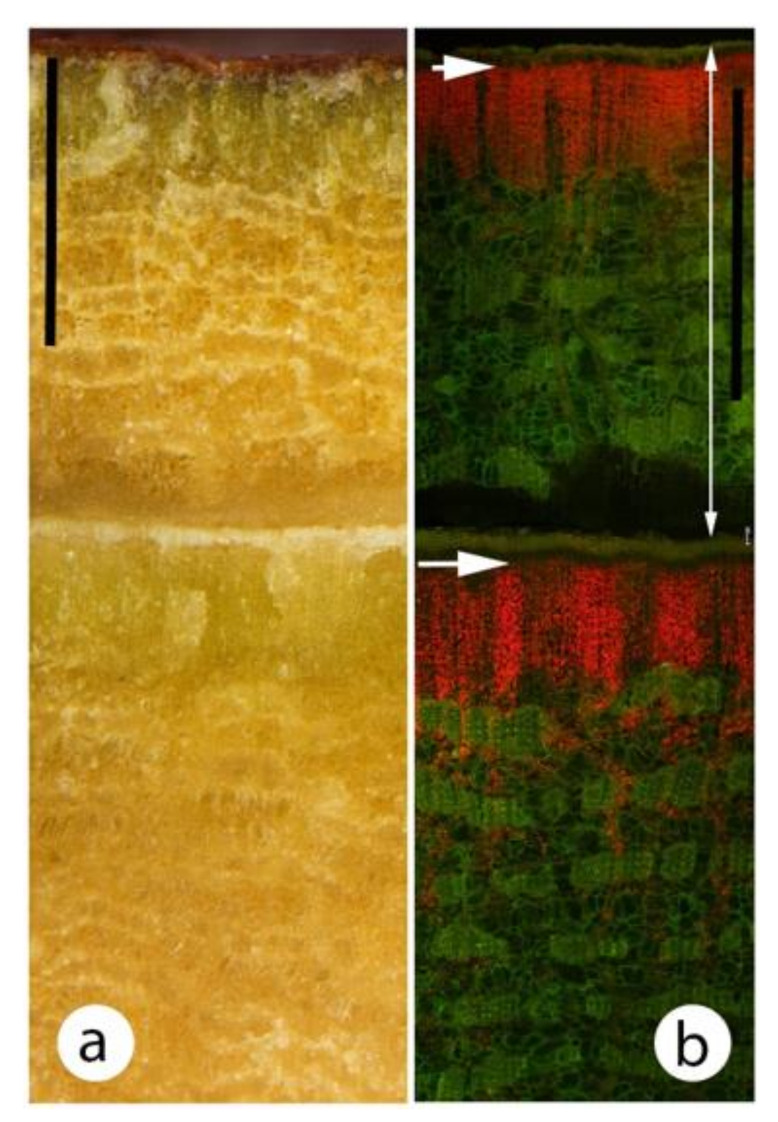
Outer bark of 40 cm diameter tree of *Corymbia citriodora* collected in November (late spring) shortly before the tree had its annual bark shedding (December). Note that a deeper, newly initiated phelloderm with a high density of chloroplasts had already been formed although outer bark abscission was still 2–4 weeks away. (**a**) Transverse plane observed using a stereo microscope with white light. Note the green band of chlorenchyma directly beneath the thin phellem. Note also an additional inner phellogen had already been initiated and an inner band of chlorenchyma had formed even though it was under 1500 μm of bark. Scale 1000 μm. (**b**) as per (**a**) but a hand-cut section showing chloroplast auto-fluorescence. short arrow, older phellogen; long arrow, recently initiated phellogen; line, layer of bark to be shed. Scale 1000 μm.

As noted, *C. citriodora* sheds its outermost bark in early summer (Figure 1c). Bark examined in late spring had a band of chlorenchyma directly below the phellem but the subsequent phellogen was already formed (800–1500 μm in from the outer older phellogen) and its phelloderm chlorenchyma layer had already formed (Figure 6). Thus two concentric bands of chlorenchyma were present and the tree would be ready to photosynthesise as soon as the outer bark was shed. When moist, the bark layer that will be shed (i.e., still living, measured immediately after being peeled from the trunk) transmitted 6% of incident radiation. When dry (e.g., brown bark on the left-hand side of Figure 1c), the bark transmitted 0.4% of incident radiation.

**Figure 7:**
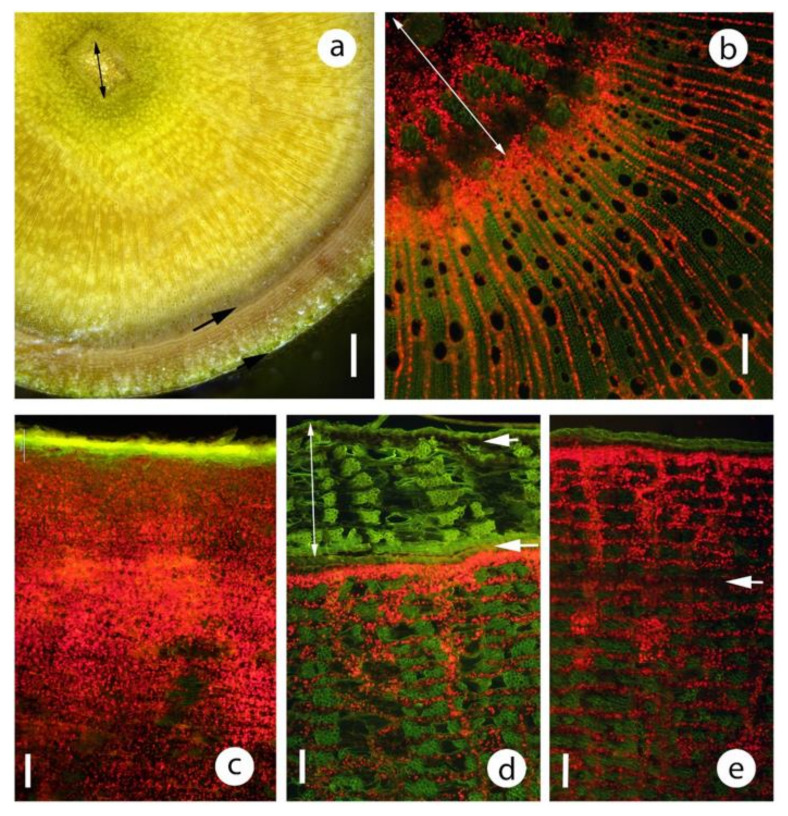
(**a**,**b**) Chloroplast distribution in an epicormic shoot of *Eucalyptus cladocalyx*, similar to that shown in Figure 1d. (**a**) transverse plane of shoot about 15 mm diameter. Note the deep green layer immediately below the epidermis, the low density of chloroplasts in the most recent secondary phloem, the greenish tint of the secondary xylem and the relatively bright green in and around the pith. short arrow, epidermis; long arrow, vascular cambium; line, pith. Scale 1000 μm. (**b**) transverse section of an 18 mm diameter shoot showing strong chlorophyll auto-fluorescence in the innermost secondary xylem and the pith. line, pith. Scale 100 μm. (**c**–**e**) *Eucalyptus caesia* stems sectioned in transverse plane, fluorescence microscopy. Scales 100 μm. (**c**) 3 cm diameter stem, similar to Figure 1d. Note that while the stem has a relatively large diameter, no periderm was present. Yellow surface layer is auto-fluorescence of a combination of the cuticle and glaucous wax layer. Note also that while the surface was highly reflective abundant chloroplasts were found to 700 μm below the cuticle. (**d**), (**e**) Similar to Figure 1b, stem 15 cm diameter. (**d**) Note that abundant chloroplasts were present below the 400 μm thick, dark brown outer bark tissues. short arrow, non-functional phellogen, long arrow, active phellogen; line, bark to be shed. (**e**) Very thin, greenish coloured bark. Note that the position of the next phellogen was apparent about 400 μm below the surface. arrow, location where next phellogen will be initiated.

The bright green *E. cladocalyx* epicormic shoots still possessed an epidermis with stomata on relatively large diameter stems (Figure 1e). Stereo microscope observation of these stems cut in a transverse plane showed the usual intense green band immediately below the epidermis (Figure 7a) but a greenish tinge was also present across the secondary xylem and a deeper green in the innermost xylem and pith (Figure 7a). Fluorescence microscopy confirmed the visual observation that chloroplasts were present all the way across the secondary xylem and were at relatively high density in the middle of the epicormic shoot (Figure 7b). Chloroplasts were recorded to a maximum depth of 10,000 μm.

Smaller diameter branches of *Eucalyptus caesia* were covered in a white waxy bloom which, when rubbed away, revealed a reddish or deep green stem (Figure 1d). These stems had not developed a periderm and, in the trees examined, were up to 6 cm in diameter. The stems, while almost a pure white with a high reflectance, still had a high chloroplast density (Figure 7c). On larger diameter stems, a minniritchi bark develops (Figure 1b). Numerous chloroplasts were present in green bark (Figure 7e) and also below the brown/red bark (dead and about 400 μm thick) (Figure 7d).

## 3. Discussion

### 3.1. General Distribution of Chloroplasts

Chloroplasts were found in the small diameter stems of all species examined and in the large diameter stems of all the smooth (‘gum’) barked species. For all species and all stem diameters, the highest concentration of chloroplasts was in a relatively narrow (usually 100–300 μm) band just inside the epidermis or phellem. While several studies (see listing in the Introduction) have noted the presence of greenish tissues in eucalypt stems, the only anatomical study with images is that of Mishra et al. [37]. They studied autofluorescence in the sapwood and heartwood of 2 and 11-year-old plants of *E. bosistoana*. Only the sapwood of the younger plants showed chlorophyll autofluorescence. The small diameter stems in the present study were obviously green, except when glaucous. Only in a couple of species (e.g., *E. caesia*) were the larger diameter stems obviously green. In the other species examined the thin phellem very effectively obscured the green tissues beneath.

### 3.2. Differences in Chloroplast Distribution Between Species

Relatively little variation was observed between species when comparing similar diameter stems. Chattaway [32] noted that the structure of twigs and young stems of eucalypts was uniform. The similarities of the outer bark of the examined species (very thin phellem, relatively thin phelloderm with numerous chloroplasts, numerous sclereids and fibres) were more notable than any minor species differences, especially when compared to the photosynthetic bark of a wide range of other Australian trees and shrubs (G. Burrows unpublished data).

One distinctive feature in the larger diameter material was that initial stereoscopic views of *C. citriodora* and *C. maculata* bark cut in transverse section indicated that these species might have had palisade-type tissue. Sectioning showed that the ‘palisade’ was columns of phelloderm cells. While this well-defined band had a relatively high percentage of chloroplast containing cells, it also had numerous thin-walled sclereids and little air space development and was thus completely unlike typical palisade tissue. For trees and shrubs, Angyalossy et al. [51] note that a thin phellogen (1–3 cell layers) is most commonly observed. At 10–15 cell layers thick, the phellogen of these two species would appear to be relatively deep.

In SNP, the stems remain green and periderm development is delayed or does not occur. In these stems, the development of palisade cells in the outer cortex has been recorded (e.g., [4,10,52,53,54]). In none of the eucalypt species examined, at none of the stem diameters sectioned, were palisade-like cells observed and none appear to have been recorded in the SRP literature.

### 3.3. Chloroplast Distribution in Small vs Large Diameter Stems

In the gum barked species, where comparisons could be made between small and large diameter stems, chloroplasts were generally found at greater depths in small diameter stems than in large diameter stems. A similar finding has been recorded in the SRP literature. For example, chloroplasts have been recorded in the secondary xylem rays or pith of small diameter stems at depths of 1500–5000 μm [6,34,42,43,44,46,48,49]. Far fewer studies have been made of larger diameter material (Table 1), with most studies indicating that the chloroplasts were in a thin band, usually 100–350 μm in depth [14,39,41,45,50]. Pilarski and Tokarz [17] investigated chloroplast distribution in both small (7 mm) and large (60 cm) diameter stems of *Fagus sylvatica*. In the former, chloroplasts were recorded to 4000 μm deep and in the latter chloroplasts were mostly to 200 μm deep, with a few scattered to 500 μm deep. This is similar to several eucalypt species in the present study with relatively deep chloroplasts in smaller diameters, and only a narrow peripheral band in larger diameters (Figure 3).

Phellem can drastically reduce light penetration into a stem. As this layer can increase in depth as a stem increases in diameter, the light transmission to the chloroplasts often reduces with increasing age of a stem. In the present study those eucalypt species with smooth bark on large diameter branches had very thin phellem thickness (almost all measurements ≤ 100 μm). In *C. citriodora,* annual bark shedding resulted in a very thin (30–50 μm) phellem on all branch diameters assessed (stems 2.5–45 cm diameter). Tausz et al. [29] reported that for *E. nitens*, for the trunk of 25 cm diameter trees, the periderm transmitted 57% of incident photosynthetically active radiation. They indicated this was “higher than normally reported” (p. 418 [29]) and the chlorenchyma had a photosynthetic pigment composition like that of sun leaves. For *C. citriodora,* the layer of bark that is shed annually, when alive, transmitted 6% of light even though it was around 800 μm thick, thus the thin phellem would probably transmit relatively high intensities into the chlorenchyma band. Rosell [15] examined outer (dead) and inner (living) bark thickness in 640 species, including 12 eucalypt species (see their Figure 3). Only about 15 species (2%) had an outer bark thickness of less than 50 μm and most of these values were for small stem diameters (e.g., <2 cm). Thus, smooth barked eucalypts in general, and *C. citriodora* in particular, would appear to have very thin phellem for trees. Eucalypts generally have pendulous leaves thus the bark receives relatively high light intensities. It may be that SRP is of greater relative importance in smooth barked eucalypts than in many other tree groups due to relatively high light intensities in the outer bark.

In the present study, the outermost layer of bark that *C. citriodora* sheds annually had a much higher light transmission when living (moist) than when it was dead (dry) and ready to be shed. It seems probable that SRP in *C. citriodora* would be reduced for a couple of weeks in early summer while this outer bark is dead but not yet shed (e.g., Figure 1c). The transverse sections of *C. citriodora* bark made in the weeks before annual bark shedding (Figure 6) indicate more than just light transmittance was involved in the distribution and/or differentiation of chloroplasts. A high density of chloroplasts was already present in the inner phelloderm layer, even though in November it still had an overlying c. 1000 μm thick layer of bark and the inner region of this overlying bark layer had few chloroplasts. These deeper phelloderm cells would appear to be predisposed to develop numerous chloroplasts, even when in a relatively low light intensity environment.

In rapidly growing epicormic shoots of *E. cladocalyx* (bright green, no periderm) (Figure 1e), relatively abundant chloroplasts were found in and near the pith of stems up to 20 mm diameter (Figure 7). How deep have chloroplasts been recorded in stems? Pfanz and Aschan [2] noted a few studies where green ‘halos’ were observed around the stem pith of woody plants. Schmitz et al. [46] found that in two *Ceriops* species with relatively thick bark, chloroplast fluorescence was only found near the pith. Van Cleve et al. [42] and Pilarski and Tokarz [17] both illustrated chloroplasts in pith cells 4000–5000 μm in from the stem surface. Thus, the 8000-10000 μm chloroplast depths found in this study are deeper but comparable to previous studies.

How do these deeply located chloroplasts receive light? Sun et al. [18], who studied the stems (0.5–3.0 cm diameter) of 21 species of woody plants, found that vessels, tracheids, and fibres conducted light efficiently in an axial direction. In vessels, the light was conducted in the lumina and in fibres and tracheids in the cell walls. They found that red/far red wavelengths were conducted most efficiently. Karabourniotis et al. [55] found that sclereids in sclerophyllous leaves of *Olea europea* act as optical fibres and may improve the light microenvironment within these relatively thick leaves. In bean seedlings, Kakuszi and Böddi [56] and Kakuszi et al. [57] recorded chlorophyll formation to 4–5 cm below the soil surface due to light piping by the hypocotyl. The information in these references does not explain why chloroplasts formed at such depths in *E. cladocalyx* but it does show that the internal light environment of stems is influenced by a complicated range of factors. The presence of chloroplasts does not necessarily mean cells are photosynthetic; chlorophyll formation can occur at light intensities below that needed for photosynthesis [58]. Deeper chloroplasts can have a starch storage function [43,49]. Further research is needed to ascertain whether the deeply located eucalypt chloroplasts are photosynthetic.

### 3.4. Stem and Leaf Stomatal Densities

In SNP species (long-lived green stems), high stem stomatal densities (50–190 stomata/mm^2^) have been recorded [4,10,59]. Young tree stems generally have either no (e.g., [6]) or low stomatal densities (e.g., [58]). A similar finding was made in the present study (average 1–4 stomata/mm^2^ with a very uneven distribution in the six species assessed). A much more intense chlorophyll fluorescence was observed in a radius of about 100 μm of the stomata. The CO_2_ concentration has been reported to be 500–800 times higher in stems or branches than ambient air [60,61]. Thus, stomata should not make any difference to internal CO_2_ concentration. However, the high concentrations referred to above are for larger diameter stems. In very young stems, there are less respiring cells, chloroplasts are present in all tissues, and thus there may be a CO_2_ shortage during the day. Higher CO_2_ concentrations have been associated with increased chloroplast density [62,63]. Most eucalypt stem chloroplasts would contribute to SRP, although a small amount of SNP probably occurs in small diameter stems in the immediate vicinity of the low density stomata.

Lenticels permit gas exchange through the otherwise impervious periderm. For *Olea europaea* and *Cercis siliquastrum,* the parenchyma cells beneath the lenticels showed a brighter red auto-fluorescence than those elsewhere [64], similar to that described for stomata in the young eucalypt branchlets. It has also been suggested that lenticels may also permit more light into the stem than the general periderm but this was not substantiated in a study of 10 species that examined one-year-old twigs [65]. Eucalypt lenticels are apparently rare. They were not observed on any of the investigated species and are rarely referred to in the literature.

### 3.5. Bark Functional Trade-Offs

Bark is a complex tissue with numerous functions (protection, storage, mechanical support, photosynthesis, translocation of photosynthates, xylem embolism repair, wound closure) [3,16]. This leads to trade-off relationships [16], e.g., protection from fire vs SRP and strength vs SRP. For eucalypts that shed bark a trade-off exists between protection from fire and maintenance of thin phellem for SRP [11]. In smooth barked eucalypts, many of the cells in prime position for photosynthesis (i.e., directly in from the phellogen, i.e., phelloderm and outermost secondary phloem or secondary cortex) would need to be fibres and sclereids for protection, e.g., in *C. citriodora,* the phelloderm had many sclereids which reduced the volume of photosynthetic tissue. In savanna ecosystems (grass, not crown fires), eucalypts with rough basal and smooth upper bark (half-barks) may have a particularly effective combination of bark types.

It would be useful to compare the bark density in the inner living bark of eucalypts with thin smooth phellem with those with thick phellem. Bark with a high proportion of thick walled sclerenchyma should have a relatively high density. We hypothesise that inner bark from species with thin phellem would be denser than inner bark that is protected by thick phellem.

### 3.6. Phellem Thickness and Chloroplast Development

Rosell et al. [3] examined several bark traits, including photosynthesis, in twigs and main stems of 85 shrub and tree species (including eight eucalypt species) in six sites from Australia and Mexico. The presence of photosynthetic bark was assessed by scraping off the rhytidome or phellem. Almost all (94%) of the twigs had photosynthetic bark, while about half (45%) of the main stems did. Their Figure 3 investigated the relationship between outer bark (i.e., dead bark/phellem) thickness and the probability of photosynthetic bark occurring in main stems. A trend existed of thin dead bark being associated with photosynthetic bark and vice versa. However, there did not appear to be a specific phellem thickness that was associated with the presence/absence of photosynthetic bark. As an example, their data indicates that about 10 species had photosynthetic bark with a dead bark thickness of >1 mm, while about 13 species had non-photosynthetic bark with an outer bark thickness of <1 mm. Other authors have noted chlorenchyma below a 1000 μm thick phellem [45] and below a 10 mm thick rhytidome [66]. In a study of relatively small diameter stems (6–13 mm) of 13 mangrove species, Schmitz et al. [46] found that xylary chlorophyll fluorescence and bark thickness (full bark thickness, not just phellem thickness) were not “strictly related” (p. 42 [46]). They also indicated that branch diameter and bark colour were not related to the distribution of xylary chloroplasts and this was found both between and within species. While the influence of phellem thickness on chloroplast depth was not directly addressed in the present study, in *E. blakelyi* (Figure 1a) and *E. caesia* (Figure 1b), chloroplasts were present beneath thicker and darker phellem.

### 3.7. Conclusions

From comparison with data in Rosell [15] it appears, on a worldwide basis, that smooth barked eucalypts may have very thin phellem. Combined with open canopies (pendulous leaves), this could mean relatively high light intensities in the chlorenchyma band in stems of all diameters and perhaps a greater importance for SRP in this group. In large diameter stems, this trade-off between maximal SRP and protection has resulted in a relatively similar outer bark anatomy in the wide diversity of species studied of: (i) thin phellem, (ii) an outer narrow band with most of the chloroplasts, and (iii) inner bark with a very high proportion of fibres and sclereids.

## 4. Materials and Methods

### 4.1. Materials

Material was sampled from 23 eucalypt species, including species of *Angophora, Corymbia,* and *Eucalyptus* and each of the three major subgenera (*Eudesmia*, *Eucalyptus*, *Symphomyrtus*) within *Eucalyptus* (Table 3). The trees sampled were mainly a mix of specimen trees and remnant vegetation on the Charles Sturt University, Wagga Wagga campus (35.06° S, 147.36° E). Thirteen of the species had smooth bark on at least the major branches and nine species had rough bark on the main stem and major branches. For species with rough bark, samples were taken from green stems where the epidermis was still present, to where the phellem had developed but was still relatively thin. For species with smooth bark throughout samples were excised from the trunk and/or main branches as well small diameter branchlets (Table 3). At least two trees per species were sampled, with at least three or four diameter sizes sampled per tree. For most of the 13 species with smooth bark on both small and large diameter stems, four to five trees were sampled and sectioned. For these species, only a single phellogen was present for most of the year. To maintain a thin layer of dead bark cells, when a new deeper phellogen was initiated, the tissues external to this meristem were soon shed.

### 4.2. Microscopy

All sections were hand cut with double-edge razor blades, then mounted in water and coverslipped (usually less than 2 h between sectioning and microscope observation). Small diameter (<8 mm) stems were sectioned whole (from epidermis to pith), medium diameter (0.8–4 cm) stems the bark, with a thin slice of secondary xylem, was sectioned and for large diameter stems (>4 cm) with >1 cm bark thickness the outer 4–5 mm of bark was examined. Most stem samples were cut in transverse section. These three diameter divisions (small, medium, large) do not always precisely correlate with the diameter bands in Table 3, especially for small and medium. The bands used in Table 3 depended on the available material and the growth stage of that material. The bark of *C. citriodora* and *E. globulus* was also sectioned in radial longitudinal and tangential longitudinal planes to further investigate the distribution of chloroplasts, fibres, and sclereids.

Chloroplast distribution was visualised with bright field and epifluorescence microscopy (Nikon Eclipse Ni microscope) using fluorescence optics (Nikon Intensilight C-HGFI) and a blue excitation filter (Nikon B-2A). Chloroplasts were identified by their bright red fluorescence. A UV filter (UV-2A) was used to examine lignified tissue (bright blue fluorescence) distribution. The depth of phellem was measured as was the depth of the band of tissue with the greatest concentration of chloroplasts. This was based on the brightest red fluorescence. In all species and all diameters, there was usually a distinctly brighter band near the stem surface. For example, in Figure 3 the bright band (greatest chloroplast density) was the following depth in these images: (a) 200–250 μm, (b) 100–150 μm, (c) 200 μm and (d) 100–150 μm. The approximate maximum depth of chloroplasts were also measured. For example, in Figure 3 the approximate maximum depths were: (a) 1800–2000 μm, (b) 1000 μm, (c) 2000 μm and (d) 700–800 μm. These measurements were estimations as discrete delimitations were not present.

### 4.3. Light Transmission

In *C. citriodora,* complete shedding of the outer bark (periderm plus some tissues beneath this) occurs over a 2–3 weeks in December in Wagga Wagga (Figure 1c). As this layer (when both dead and alive) can be easily collected in relatively large sheets, this provided a way to quantify the influence of the outermost bark on light intensity reaching inner regions of the stem. Transmission of light through bark that was soon to be shed but still alive (moist) was compared to bark that had already been shed (on the ground below trees, naturally dried). A Li-Cor quantum sensor, connected to a Li-Cor LI-185B, was placed perpendicular to the sun’s rays (1600 μmol m^−2^ s^−1^), and sheets of the two bark types were placed over the sensor and light transmission measured.

### 4.4. Maximum Chloroplast Depth

Most eucalypts begin to develop periderm on relatively small diameter stems (e.g., 4–10 mm diameter). Some eucalypt species (e.g., *E. caesia*, *E. cladocalyx*) had relatively large diameter stems (up to 3 cm) that had no periderm development (Figure 1d,e). For *E. cladocalyx,* this development was best expressed on vigorous epicormic shoots that formed after tree damage (storms, severe pruning) (Figure 1e). When these epicormic shoots were cut with secateurs in a transverse plane, the exposed surface appeared greenish from the epidermis to the pith. These shoots were sectioned to determine if chloroplasts were present from immediately below the epidermis to the pith. For *E. caesia,* the development of relatively large diameter shoots that had not commenced periderm development was a normal part of stem development. These shoots were usually distinctly glaucous (Figure 1d). These stems were sectioned to determine if the thick waxy and highly reflective layer reduced chloroplast development.

### 4.5. Stomatal Density in Stems and Leaves

Small green areas (e.g., Figure 2f) were observed with a stereo microscope on the small diameter stems of most species. They appeared to be associated with stomata and thus stomatal density (stomata/mm^2^) was measured on small diameter stems that had not begun bark formation and also for leaves from adjoining nodes for six species (Table 2). For leaves, clear nail polish was applied to abaxial and adaxial surfaces, allowed to harden, peeled off, mounted on glass slides, photographed under a compound microscope, then stomata were counted (3 positions × 3 leaves × 2 plants). Stems were observed with a stereo microscope (Nikon SMZ25), images taken (e.g., Figure 2e) and number of stomata counted in 1 × 3 mm areas (3 positions × 2 stems × 2–3 plants).

## Figures and Tables

**Table 1:** Anatomical studies of stem structure and chloroplast distribution relevant to stem recycling photosynthesis in woody plants. Studies are arranged in chronological order.

Author	Date	Species	Materials Examined
Scott [38]	1907	30 woody species	Young shoots and inflorescence axis
Sokolov [33]	1953	100 angiosperm spp., 16 gymnosperm spp.	
Pearson & Lawrence [39]	1958	*Populus tremuloides*	19 yr old, 16 cm diameter at 2 m above ground level
Gómez-Vazquez & Engleman [40]	1984	*Bursera longipes*, *B. copallifera*	Trees 8 and 5 m high, respectively (no diameter given)
Kauppi [41]	1991	*Betula pendula*, *B. pubescens*	0, 1, 3, 4, 5, 10 and 20 yr old branches
van Cleve et al. [42]	1993	*Populus* × *canadensis*	2–3 yr old twigs, 1–2 cm diameter
Pilarski [43]	1999	*Syringa vulgaris*	Current year, 1 and 3 yr old stems
Pilarski & Tokarz [17]	2006	*Fagus sylvatica*	2 and 6 yr old stems and trunk (3.4, 6.9 and 600 mm diameter, respectively)
Dima et al. [34]	2006	20 species	2 yr old twigs
Berveiller et al. [44]	2007	9 tree species	Current year stems, 2-5 mm diameter
Filippou et al. [5]	2007	*Olea europaea*	1–30 yr old stems
Rentzou & Psaras [6]	2008	3 Mediterranean species	1–2 yr old and 2–5 yr old twigs
Kotina et al. [45]	2012	14 species Apiaceae	Branch tips to thicker stems with mature bark
Schmitz et al. [46]	2012	13 Australian mangrove species	Mainly 6 mm diameter (range 2.8–14 mm diameter)
Cocoletzi et al. [47]	2013	*Myriocarpa longipes*, *Urera glabriuscula*	10 yr old trees
Kocurek et al. [48]	2015	*Clusia* multiflora, *C. rosea*	2.0–2.5 cm diameter at 1 m above ground level
Wittmann & Pfanz [49]	2016	5 woody species	Current year shoots, 3–5 mm diameter
Kotina et al. [14]	2017	*Adansonia digitata*	Young twigs to stems 12–17 cm diameter
Blagitz et al. [50]	2019	*Monquinastrum polymorphum* *Zanithoxylum rhoifolium*	Large diameter stems

**Table 2:** Leaf and small diameter stem average stomatal densities (number/mm^2^ ± SD) for six eucalypt species.

Species	Leaf (Adaxial)	Leaf (Abaxial)	Stem
*Corymbia citriodora*	267 (± 28)	320 (±39)	2.3 (± 1.2)
*Corymbia ficifolia*	0 (± 0)	209 (±39)	1.6 (± 0.8)
*Eucalyptus cladocalyx*	0 (± 0)	279 (±27)	1.9 (± 0.8)
*Eucalyptus melliodora*	184 (± 31)	194 (±60)	4.1 (± 2.1)
*Eucalyptus sideroxylon*	134 (± 17)	169 (±22)	1.3 (± 1.1)
*Eucalyptus torquata*	105 (± 24)	124 (±26)	2.2 (± 1.6)

**Table 3:** Species studied and various anatomical measurements. Subgenera and sections are from Nicolle, D. (2019) Classification of the eucalypts (*Angophora, Corymbia* and *Eucalyptus*) Version 4 http://www.dn.com.au/ Bark types and species distribution (states or territories) are from an online version of the EUCLID Eucalypts of Australia 4th Edition (2015). A ‘-‘ in ‘phellem thickness’ indicates a periderm was not present (epidermis and cuticle still present). Stem diameter: small (S) < 8 mm, medium (M) 8–40 mm, large (L) > 4 cm.

Genus/Species	State/Territory	Subgenus-Section	Bark Type	Stem Diameter	Phellem Thickness (μm)	Chlorenchyma Bright Band (μm)	Chlorenchyma Maximum Depth (μm)
*Angophora*							
*A. hispida* (Sm.) Blaxell	NSW		Rough to small branches, fibrous	S (2 mm) S (6–8 mm) M (13–15 mm)	- 30–70 30–60	100–200 150–200 200	600–1000 800–1000 1700–1800
*Corymbia*							
*C. citriodora* (Hook.) K.D. Hill & L.A.S. Johnson	Qld	*Blakella*	Smooth throughout—gum	S (1.5–3 mm) S/M (5–11 mm) L (10–40 cm)	- 50–70 20–90	150–200 100–150 100–330	1000–1400 500–1500 700–1300
*C. maculata* (Hook.) K.D. Hill & L.A.S. Johnson	NSW, Qld, Vic	*Blakella*	Smooth throughout	S (3–6 mm) M (11–14 mm) L (30–50 cm)	0–60 30–40 30–50	150–200 120–150 120–200	1000 1400–1500 900–1800
*C. ficifolia* (F. Muell.) K.D. Hill & L.A.S. Johnson	WA	*Corymbia*	Rough to small branches, fibrous	S (3–6 mm) M (14–15 mm) M (26 mm)	- 50–100 50–80	220–400 250–300 300	1200–2000 1800–2100 1800–2100
*C. eximia* (Schauer) K.D. Hill & L.A.S. Johnson	NSW	*Blakella*	Rough to small branches; tessellated, flaky	S (2–7 mm) M (10–30 mm) L (7–10 cm)	- 50–100 100	200–400 100–400 100–120	1100–2000 1000–3000 1100–1400
*Eucalyptus*							
*E. erythrocorys* F. Muell.	WA	*Eudesmia*	Smooth; can have rough bark on lower trunk	L (4–5 cm) L (15–20 cm)	50–80 60–100	80–100 100	2300–3000 2500–2800
*E. macrorhyncha* F. Muell. ex Benth.	NSW, Vic, SA	*Eucalyptus*	Rough to small branches (stringybark)	S (2 mm) M (8–18 mm)	- 20–100	120–150 120–220	900–1000 1000–3000
*E. rossii* R.T. Baker & H. G. Sm.	NSW	*Eucalyptus*	Smooth (scribbly gum)	S (2 mm) M (11–20 mm) L (10–20 cm)	- 30–80 30–100	120–150 200–400 150–280	900–1000 1500–2000 1000–1300
*E. leucoxylon* F. Muell.	SA, Vic	*Symphyomyrtus—Adnataria*	Smooth throughout, sometimes with rough box-type at base	S (1.5–4.5 mm) S (7 mm) L (17 cm)	- 20 80–90	170–200 150 150–300	800–2000 1300 700–1100
*E. melliodora* A. Cunn. ex Schauer	NSW, Vic, Qld	*Symphyomyrtus—Adnataria*	Rough box-type at base, smooth higher	S (2.5–3 mm) S (6–8 mm) L (50–80 cm)	- 30–50 50–100	150–200 150–200 100–150	1100–1500 700–1400 700–800
*E. sideroxylon* A. Cunn. ex Woolls	NSW, Vic, Qld	*Symphyomyrtus—Adnataria*	Rough throughout (ironbark)	S (3–8 mm) M (10–12 mm) M (23–26 mm)	- 30–70 40–60	200 150–250 100–200	1000–1900 1800–2200 800–2300
*E. albens* Benth.	NSW, Vic, Qld, SA	*Symphyomyrtus—Adnataria*	Rough on trunk and large branches (box-type)	S (2–4 mm) M (12–28 mm)	- 50–80	250–400 150–350	700–1900 900–1500
*E. caesia* Benth.	WA	*Symphyomyrtus—Bisectae*	Minnirichi on trunk and branches	*1			
*E. macrocarpa* Hook.	WA	*Symphyomyrtus—Bisectae*	Smooth	S/M (3–15 mm) L (8–10 cm)	- 40–50	200–500 100–200	2000–3200 800–1100
*E. kruseana* F. Muell.	WA	*Symphyomyrtus—Glandulosae*	Rough but thin on trunk base, smooth above	S (3–8 mm) M (15–40 mm)	- 100	200–300 50–100	1000–2600 500–1500
*E. torquata* Luehm.	WA	*Symphyomyrtus—Dumaria*	Rough, hard, shortly fibrous to almost tessellated	S (3–8 mm) M (15–30 mm)	- 100–300	250–400 100–300	1000–3500 500–1400
*E. blakelyi* Maiden	NSW, Vic, Qld	*Symphyomyrtus—Exsertaria*	Smooth throughout (gum)	S (3–7 mm) M (10–40 mm) L (20–37 cm)	- 20–60 30–100	200–300 100–300 100–200	1200–1600 1500–2000 300–700
*E. camaldulensis* Dehnh.	Most states	*Symphyomyrtus—Exsertaria*	smooth	S (3–6 mm) M (8–25 mm) L (6–80 cm)	- 30–50 30–70	150–200 100–200 100–200	900–1800 500–800 500–800
*E. globulus* Labill.	Tas, Vic	*Symphyomyrtus—Maidenaria*	Smooth apart from the base with persistent slabs (gum)	S (3–8 mm) M (18–20 mm) L (50–60 cm)	- 30–40 30–80	200–300 150–200 150–300	1300–2800 1000–1800 800–1200
*E. cinerea* F. Muell. ex Benth.	NSW	*Symphyomyrtus—Maidenaria*	Thick, furrowed, fibrous to small branches	S (2–6 mm) M (10–20 mm)	- 10–80	150–200 100–250	900–2700 1500–3500
*E. nicholii* Maiden & Blakely	NSW	*Symphyomyrtus—Maidenaria*	Thick, fibrous to small branches (peppermint)	S (3–4 mm) M (8–18 mm)	- 20–50	150–200 200–350	1300–1900 1700–2300
*E. scoparia* Maiden	Qld	*Symphyomyrtus—Maidenaria*	Smooth—gum	S (3–8 mm) L (4–26 cm)	- 30–50	150–300 150–400	1400–2800 900–1300
*E. cladocalyx* F. Muell.	SA	*Symphyomyrtus—Sejunctae*	Smooth—gum	S (2–8 mm) L (24–60 cm)	0–60 40–70	180–300 80–150	800–3900 600–800

*1 see Section 2 for detail.
